# The genome sequence of the European nightjar, *Caprimulgus europaeus* (Linnaeus, 1758)

**DOI:** 10.12688/wellcomeopenres.17451.1

**Published:** 2021-12-07

**Authors:** Simona Secomandi, Fernando Spina, Giulio Formenti, Guido Roberto Gallo, Manuela Caprioli, Roberto Ambrosini, Sara Riello, Wellcome Sanger Institute Tree of Life programme, Wellcome Sanger Institute Scientific Operations: DNA Pipelines collective, Tree of Life Core Informatics collective, Darwin Tree of Life Consortium

**Affiliations:** 1 Department of Biosciences, University of Milan, Milan, Italy; 2 Institute for Environmental Protection and Research (ISPRA), Ozzano dell'Emilia, Italy; 3 Vertebrate Genome Laboratory, The Rockefeller University, New York, NY, USA; 4 Howard Hughes Medical Institute, Chevy Chase, MD, USA; 5 Department of Environmental Sciences and Policy, University of Milan, Milan, Italy; 6 Riserva Naturale Statale “Isole di Ventotene e S. Stefano”, Ventotene, Italy

**Keywords:** Caprimulgus europaeus, European nightjar, Eurasian nightjar, genome sequence, chromosomal

## Abstract

We present a genome assembly from an individual female *Caprimulgus europaeus *(the European nightjar; Chordata; Aves; Caprimulgiformes; Caprimulgidae). The genome sequence is 1,178 megabases in span. The majority of the assembly (99.33%) is scaffolded into 37 chromosomal pseudomolecules, including the W and Z sex chromosomes.

## Species taxonomy

Eukaryota; Metazoa; Chordata; Craniata; Vertebrata; Euteleostomi; Archelosauria; Archosauria; Dinosauria; Saurischia; Theropoda; Coelurosauria; Aves; Neognathae; Caprimulgimorphae; Caprimulgiformes; Caprimulgidae; Caprimulginae; Caprimulgus; *Caprimulgus europaeus* Linnaeus 1758 (NCBI:txid85660).

## Background

The European nightjar ( *Caprimulgus europaeus*; also known as the Eurasian nightjar and common goatsucker) is an insectivorous, crepuscular, ground-nesting bird distributed throughout the Western Palearctic ( Hagemeijer & Blair, 1997). It breeds in semi-natural dry and open habitats with scattered trees ( Cramp & Brooks, 1985). Little is known about the ecology of the European nightjar ( Cramp & Brooks, 1985; Polakowski *et al*., 2020), and in general that of the Caprimulgidae family. The family comprises peculiar species such as the only bird known to hibernate, the Common Poorwill ( *Phalaenoptilus nuttallii*) ( Carey, 2019; French, 2019; Woods *et al.*, 2019), and one of the few birds that uses echo-localization, the South American Oilbird ( *Steatornis caripensis*) ( Brinkløv *et al.*, 2013). The European nightjar has been found to be more resistant to pathogens than other bird species ( Jiang *et al*., 2021). Although categorized as ‘least concern’ by the IUCN ( IUCN, 2016), the European nightjar has experienced a steady population decline in the past decades, and is of conservation concern in Europe ( Eaton *et al*., 2015; Evens *et al*., 2017; Keller *et al*., 2010). The availability of a high-quality, chromosome-level reference genome will help to deepen the knowledge on the biology and evolution of this species, boosting studies on the genomics of the peculiar family of Caprimulgidae. Moreover, as genomic resources gain preheminence in conservation efforts ( Allendorf, 2017; Fuentes-Pardo & Ruzzante, 2017; Supple & Shapiro, 2018), we expect that the reference genome presented here will help aid planning conservation actions for the European nightjar.

## Genome sequence report

The genome was sequenced from a blood sample taken from a single female *C. europaeus* collected from a bird ringing station in Ventotene, Italy (latitude 40.79404, longitude 13.42777). A total of 87-fold coverage in Pacific Biosciences single-molecule long reads and 62-fold coverage in 10X Genomics read clouds were generated. Primary assembly contigs were scaffolded with chromosome conformation Hi-C data. Manual assembly curation corrected 144 missing/misjoins and removed 31 haplotypic duplications, reducing the assembly length by 0.15% and the scaffold number by 21.94%, and increasing the scaffold N50 by 26.46%.

**Figure 1.**
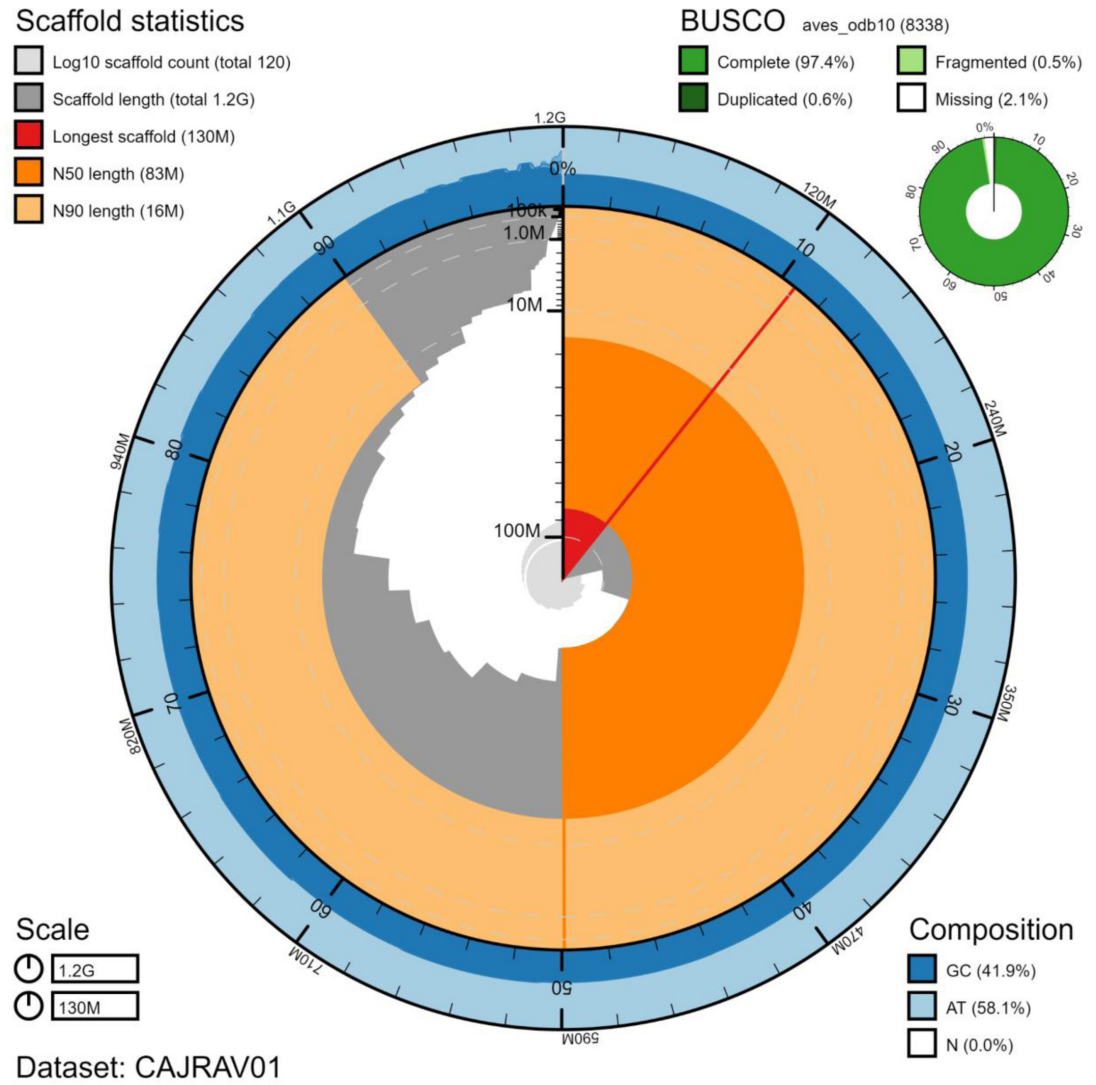
Genome assembly of *Caprimulgus europaeus*, bCapEur3.1: metrics. The BlobToolKit Snailplot shows N50 metrics and BUSCO gene completeness. The main plot is divided into 1,000 size-ordered bins around the circumference with each bin representing 0.1% of the 1,177,791,212 bp assembly. The distribution of chromosome lengths is shown in dark grey with the plot radius scaled to the longest chromosome present in the assembly (126,318,510 bp, shown in red). Orange and pale-orange arcs show the N50 and N90 chromosome lengths (82,614,289 and 15,699,869 bp), respectively. The pale grey spiral shows the cumulative chromosome count on a log scale with white scale lines showing successive orders of magnitude. The blue and pale-blue area around the outside of the plot shows the distribution of GC, AT and N percentages in the same bins as the inner plot. A summary of complete, fragmented, duplicated and missing BUSCO genes in the aves_odb10 set is shown in the top right. An interactive version of this figure is available at https://blobtoolkit.genomehubs.org/view/bCapEur3.1/dataset/CAJRAV01/snail.

The final assembly has a total length of 1,178 Mb in 121 sequence scaffolds with a scaffold N50 of 83 Mb ( Table 1). Of the assembly sequence, 99.3% was assigned to 37 chromosomal-level scaffolds, representing 35 autosomes (numbered by sequence length) and the W and Z sex chromosomes ( Figure 1– Figure 4; Table 2). The assembly has a BUSCO ( Simão *et al*., 2015) completeness of 97.4% (single 96.9%, duplicated 0.6%) using the aves_odb10 reference set. While not fully phased, the assembly deposited is of one pseudo-haplotype. Contigs corresponding to the alternate haplotype have also been deposited.

**Table 1.** Genome data for *Caprimulgus europaeus*, bCapEur3.1.

*Project accession data*	
Assembly identifier	bCapEur3.1
Species	*Caprimulgus europaeus*
Specimen	bCapEur3
NCBI taxonomy ID	NCBI:txid111811
BioProject	PRJEB44540
BioSample ID	SAMEA7524394
Isolate information	Female, blood
*Raw data accessions*	
PacificBiosciences SEQUEL II	ERR6445211
10X Genomics Illumina	ERR6054683-ERR6054686
Hi-C Illumina	ERR6054687, ERR6054688
*Genome assembly*	
Assembly accession	GCA_907165065.1
*Accession of alternate haplotype*	GCA_907165095.1
Span (Mb)	1,178
Number of contigs	274
Contig N50 length (Mb)	31
Number of scaffolds	121
Scaffold N50 length (Mb)	83
Longest scaffold (Mb)	126
BUSCO * genome score	C:97.4%[S:96.9%, D:0.6%],F:0.5%,M:2.1%,n:8338

*BUSCO scores based on the aves_odb10 BUSCO set using v5.1.2. C= complete [S= single copy, D=duplicated], F=fragmented, M=missing, n=number of orthologues in comparison. A full set of BUSCO scores is available at https://blobtoolkit.genomehubs.org/view/bCapEur3.1/dataset/CAJRAV01/busco.

**Figure 2.**
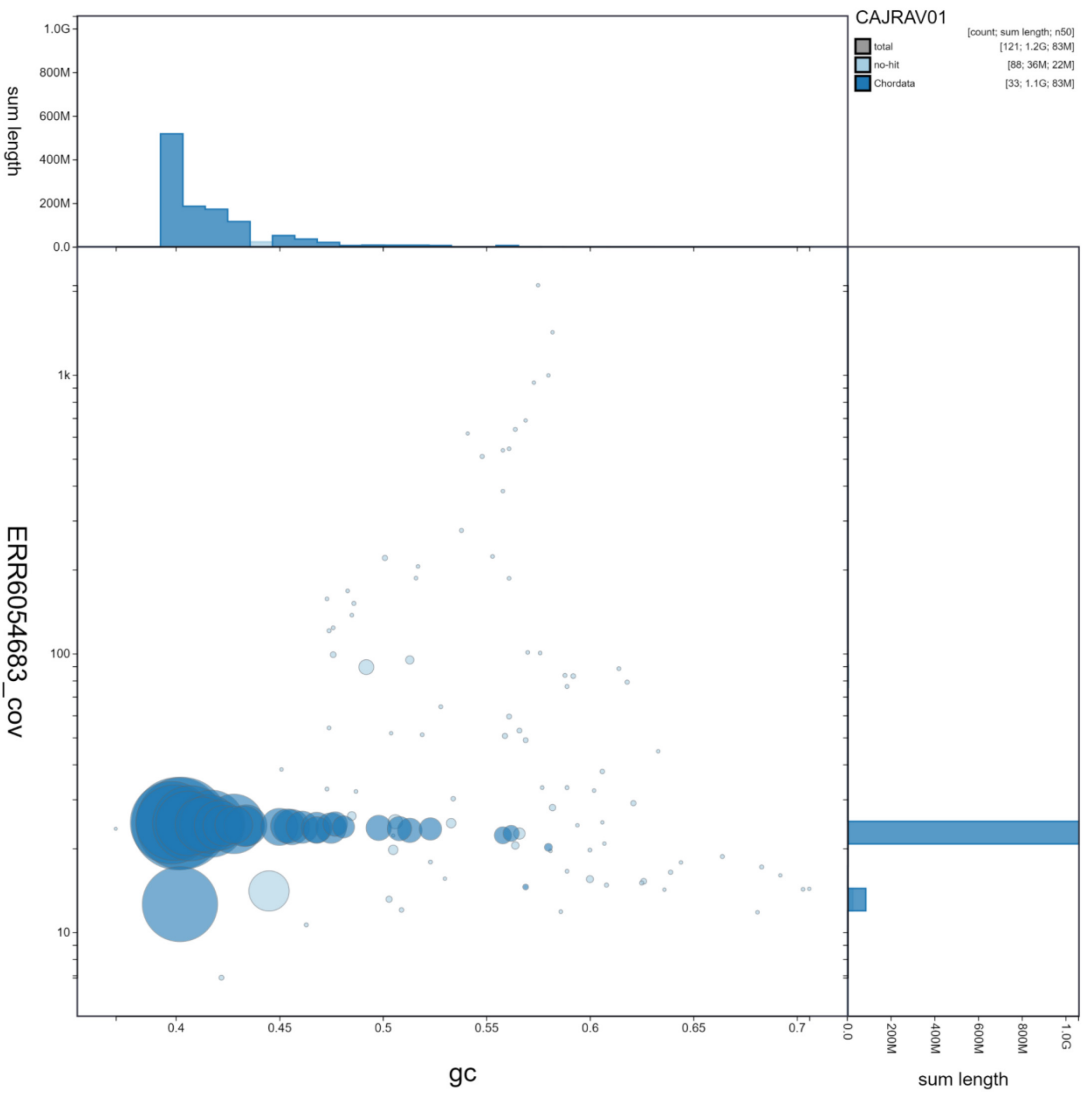
Genome assembly of *Caprimulgus europaeus*, bCapEur3.1: GC coverage. BlobToolKit GC-coverage plot. Scaffolds are coloured by phylum. Circles are sized in proportion to scaffold length. Histograms show the distribution of scaffold length sum along each axis. An interactive version of this figure is available at https://blobtoolkit.genomehubs.org/view/bCapEur3.1/dataset/CAJRAV01/blob.

**Figure 3.**
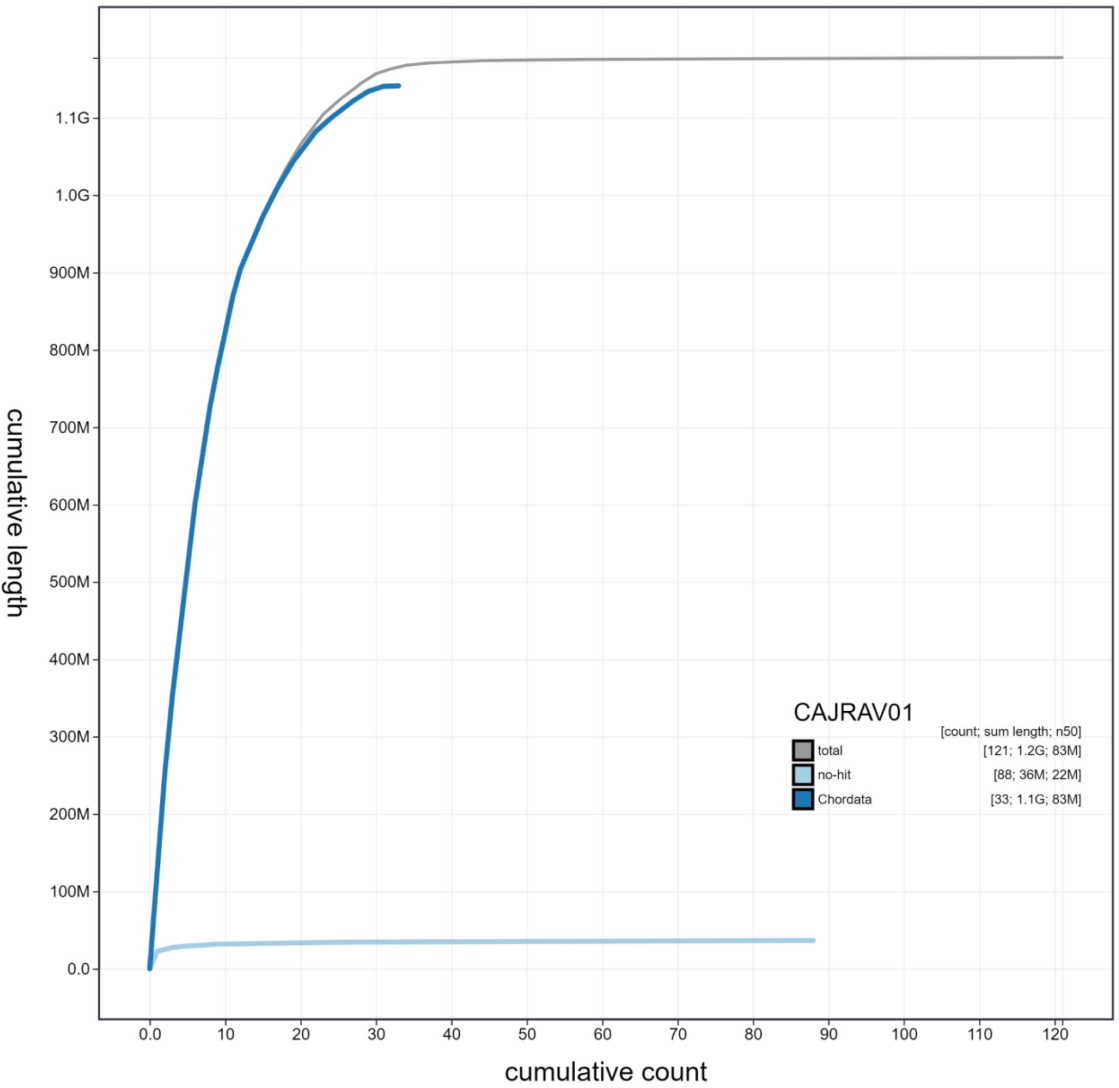
Genome assembly of *Caprimulgus europaeus*, bCapEur3.1: cumulative sequence. BlobToolKit cumulative sequence plot. The grey line shows cumulative length for all scaffolds. Coloured lines show cumulative lengths of scaffolds assigned to each phylum using the buscogenes taxrule. An interactive version of this figure is available at https://blobtoolkit.genomehubs.org/view/bCapEur3.1/dataset/CAJRAV01/cumulative.

**Figure 4.**
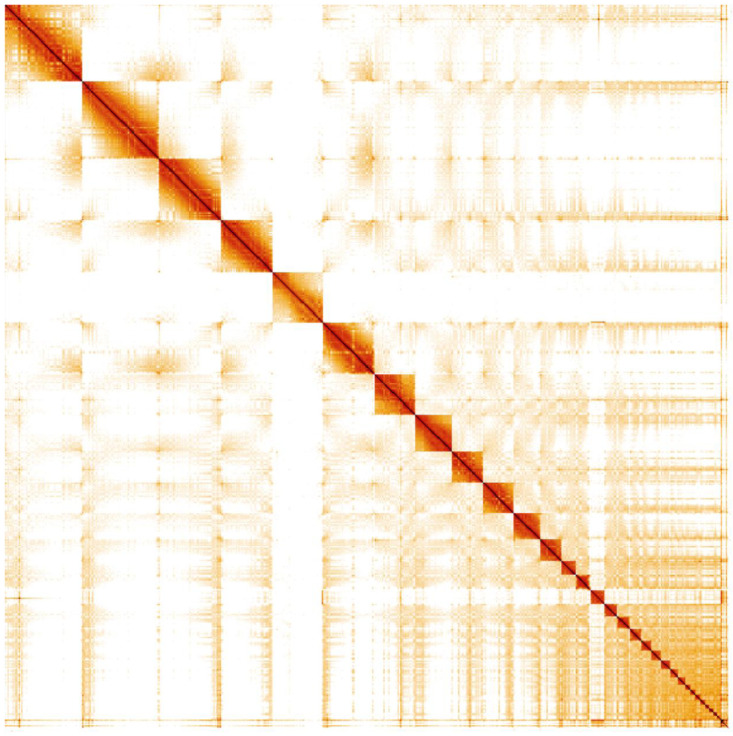
Genome assembly of *Caprimulgus europaeus*, bCapEur3.1: Hi-C contact map. Hi-C contact map of the bCapEur3 assembly, visualised in HiGlass. Chromosomes are shown in order of size from left to right and top to bottom.

**Table 2.** Chromosomal pseudomolecules in the genome assembly of *Caprimulgus europaeus*, bCapEur3.1.

INSDC accession	Chromosome	Size (Mb)	GC%
OU015523.1	1	126.32	40.1
OU015524.1	2	125.37	40.3
OU015525.1	3	100.16	39.8
OU015526.1	4	83.32	39.9
OU015528.1	5	82.61	40.7
OU015529.1	6	65.35	41.7
OU015530.1	7	60.47	40.6
OU015531.1	8	50.91	42.8
OU015532.1	9	48.66	41.6
OU015533.1	10	43.00	41.3
OU015534.1	11	35.23	42.1
OU015535.1	12	23.52	43.4
OU015536.1	13	22.81	42.3
OU015538.1	14	22.35	43.3
OU015539.1	15	19.40	42.8
OU015540.1	16	18.74	45
OU015541.1	17	16.93	45.6
OU015542.1	18	15.70	45.4
OU015543.1	19	13.78	46.1
OU015544.1	20	12.52	46.8
OU015545.1	21	12.35	47.5
OU015546.1	22	9.16	46.8
OU015547.1	23	8.19	49.8
OU015548.1	24	7.57	47.7
OU015549.1	25	7.54	51.3
OU015550.1	26	7.50	50.8
OU015551.1	27	6.26	52.3
OU015552.1	28	6.04	48.1
OU015553.1	29	3.39	55.8
OU015554.1	30	2.94	56.1
OU015555.1	31	2.47	49.2
OU015556.1	32	2.22	50.6
OU015557.1	33	1.26	56.6
OU015558.1	34	0.56	51.3
OU015559.1	35	0.20	47.7
OU015537.1	W	22.49	44.5
OU015527.1	Z	82.63	40.2
-	Unplaced	7.86	54.9

## Methods

### Sample acquisition

Sampling was performed during the routine activity of the scientific ringing station located in Ventotene island, Latina, Italy (latitude 40.7926°, longitude 13.4241°) during spring migration. Samples have been collected by ISPRA researchers within their institutional activities as from Italian national Law n. 157/92. Bird capture was performed in the evening according to standardized protocols using mist-nets ( Saino *et al*., 2010; Spina *et al.*, 1993). The sample was collected with a heparinized capillary tube after puncturing the ulnar vein with an intra-epidermal needle. The blood was immediately transferred into 99% ethanol, initially kept at room temperature and then frozen.

### DNA extraction and sequencing

High molecular weight DNA was extracted from the blood sample at the Scientific Operations core of the Wellcome Sanger Institute using the Bionano Prep Blood DNA Isolation Kit according to the Bionano Prep Frozen Blood protocol. Pacific Biosciences CLR long read and 10X Genomics read cloud sequencing libraries were constructed according to the manufacturers’ instructions. Sequencing was performed by the Scientific Operations core at the Wellcome Sanger Institute on Pacific Biosciences SEQUEL II and Illumina HiSeq X instruments. Hi-C data were generated from the same blood sample using the Arima Hi-C+ kit and sequenced on HiSeq X.

### Genome assembly

Assembly was carried out following the Vertebrate Genome Project pipeline v1.6 ( Rhie *et al*., 2020) with Falcon-unzip ( Chin *et al*., 2016), haplotypic duplication was identified and removed with purge_dups ( Guan *et al*., 2020) and a first round of scaffolding carried out with 10X Genomics read clouds using scaff10x. Scaffolding with Hi-C data ( Rao *et al*., 2014) was carried out with SALSA2 ( Ghurye *et al*., 2019). The Hi-C scaffolded assembly was polished with arrow using the PacBio data, with merfin ( Formenti *et al*., 2021b) applied to avoid a drop in QV, then polished with the 10X Genomics Illumina data by aligning to the assembly with longranger align, calling variants with freebayes ( Garrison & Marth, 2012) and applying homozygous non-reference edits using bcftools consensus. A complete mitochondrion was not found using mitoVGP ( Formenti *et al*., 2021a), likely due to the sample being sourced from blood tissue, so mitochondrial sequence NC_025773.1 ( *Caprimulgus indicus*) was used during polishing. The assembly was checked for contamination and corrected using the gEVAL system ( Chow *et al*., 2016) as described previously ( Howe *et al*., 2021). Manual curation ( Howe *et al*., 2021) was performed using gEVAL, HiGlass ( Kerpedjiev *et al*., 2018) and Pretext. The genome was analysed, and BUSCO scores generated, within the BlobToolKit environment ( Challis *et al*., 2020). Table 3 gives version numbers of the software tools used in this work.

**Table 3.** Software tools used.

Software tool	Version	Source
Falcon-unzip	1.8.0	Chin *et al*., 2016
purge_dups	1.2.3	Guan *et al*., 2020
SALSA2	2.2	Ghurye *et al*., 2019
Arrow	GCpp-1.9.0	https://github.com/PacificBiosciences/GenomicConsensus
Merfin	1.7	Formenti *et al*., 2021b
longranger align	2.2.2	https://support.10xgenomics.com/genome-exome/software/pipelines/latest/advanced/other-pipelines
freebayes	1.3.1-17-gaa2ace8	Garrison & Marth, 2012
gEVAL	N/A	Chow *et al*., 2016
HiGlass	1.11.6	Kerpedjiev *et al*., 2018
PretextView	0.1.x	https://github.com/wtsi-hpag/PretextView
BlobToolKit	2.6.2	Challis *et al*., 2020

## Data availability

European Nucleotide Archive: Caprimulgus europaeus (Eurasian nightjar). Accession number PRJEB44830; https://identifiers.org/ena.embl:PRJEB44830.

The genome sequence is released openly for reuse. The *C. europaeus* genome sequencing initiative is part of the Darwin Tree of Life (DToL) project and the Vertebrate Genomes Project. All raw sequence data and the assembly have been deposited in INSDC databases. The genome will be annotated and presented through the Ensembl pipeline at the European Bioinformatics Institute. Raw data and assembly accession identifiers are reported in Table 1.

## Author information

Members of the Darwin Tree of Life Consortium are listed here: https://doi.org/10.5281/zenodo.4783559.

Members of the Darwin Tree of Life Barcoding collective are listed here: https://doi.org/10.5281/zenodo.4893704.

Members of the Wellcome Sanger Institute Tree of Life collective are listed here: https://doi.org/10.5281/zenodo.4783586.

Members of the Sanger Scientific Operations: DNA Pipelines collective are listed here: https://doi.org/10.5281/zenodo.4790456.

Members of the Tree of Life Core Informatics collective are listed here: https://doi.org/10.5281/zenodo.5013542.
