# Supplementary material for: The genome sequence of the European nightjar, Caprimulgus europaeus (Linnaeus, 1758)
Source: Wellcome Open Res. 2021 Dec 7;6:332. [Version 1] doi: 10.12688/wellcomeopenres.17451.1 (PMC8729189; doi:10.12688/wellcomeopenres.17451.1)
Supplement: Peer review history

# Peer review: Reviewer response for version 1

**DOI:** 10.21956/wellcomeopenres.19297.r47480

**Published:** 2022-01-04

**Authors:** Joshua Peñalba

**Affiliations:** 1 Center for Integrative Biodiversity Discovery, Museum für Naturkunde, Berlin, Germany

The authors describe the sequencing and assembly of the chromosome-scale reference genome for the European Nightjar. The methods follow that of the Vertebrate Genome Project pipeline. I just have some minor comments:

- How was the bird identified as female?
- About how much blood was used for the sequencing?
- How was the quality of the DNA checked?
- How many PacBio cells and Illumina lanes were used for each sequencing method?
- How did you know how many chromosomes should have been assembled?
- Can you provide more details on the assembly, which parameters were used and how was manual curation performed? If this is detailed in a different manuscript, please explicitly state which manuscript.

Are sufficient details of methods and materials provided to allow replication by others?

Partly

Is the rationale for creating the dataset(s) clearly described?

Yes

Are the datasets clearly presented in a useable and accessible format?

Yes

Are the protocols appropriate and is the work technically sound?

Yes

Reviewer Expertise:

genomics, evolution, population genomics

I confirm that I have read this submission and believe that I have an appropriate level of expertise to confirm that it is of an acceptable scientific standard.

# Peer review: Reviewer response for version 1

**DOI:** 10.21956/wellcomeopenres.19297.r47481

**Published:** 2021-12-20

**Authors:** Anne-Lyse Ducrest

**Affiliations:** 1 Department of Ecology and Evolution, University of Lausanne, Lausanne, Switzerland

The authors described a nice almost complete genome with pseudo-chromosomes of the European nightjar using PacBio Sequel II, Illumina, and HiCi sequencing methods and thus present important data for further genetic analysis.

 There are two points that could be improved:

- There are some redundancies between Figures 1, 2, and Table 1.
- The method how to get long HMV DNA is not well described since the Bionano protocol is for human blood and not for bird blood.

Are sufficient details of methods and materials provided to allow replication by others?

Partly

Is the rationale for creating the dataset(s) clearly described?

Yes

Are the datasets clearly presented in a useable and accessible format?

Yes

Are the protocols appropriate and is the work technically sound?

Yes

Reviewer Expertise:

genomic, molecular biology

I confirm that I have read this submission and believe that I have an appropriate level of expertise to confirm that it is of an acceptable scientific standard.
